# Examining the impact of early longitudinal patient exposure on medical students’ career choices

**Published:** 2017-02-24

**Authors:** Jason Kwok, Vincent Wu, Anthony Sanfilippo, Kathryn Bowes, Sheila Pinchin

**Affiliations:** 1 Undergraduate Medical Education, Faculty of Health Sciences, Queen’s University, Ontario, Canada

## Abstract

**Background:**

Medical schools include career direction experiences to help students make informed career decisions. Most experiences are short, precluding students from attaining adequate exposure to long-term encounters within medicine. We investigated the impact of the First Patient Program (FPP), which fosters longitudinal patient exposure by pairing junior medical students with chronically ill patients through their healthcare journey, in instilling career direction.

**Methods:**

Medical students who completed at least 6-months in the FPP participated in a cross-sectional survey. Students’ answers were analyzed with respect to the number of FPP appointments attended. Thematic analysis was conducted to explore qualitative responses.

**Results:**

One hundred and forty-eight students participated in the survey. Only 28 (19%) students stated that the FPP informed their career decisions. Thirty-nine percent of students who attended four or more appointments indicated that the FPP informed their career decisions, compared to 16% of students who attended less (p=0.021). Thematic analysis revealed two themes: 1) Students focused mainly on patient encounters within FPP; and 2) Students sought career directions from other experiences.

**Conclusion:**

The majority of students did not attain career guidance from the FPP, but rather used the program to understand the impact of chronic illness from the patient’s perspective.

## Introduction

Making informed career choices is arguably one of the most important decisions medical students must make during their journey through medical school. The importance is highlighted within the CANMEDs 2015 Series II draft framework as part of the Leader competency role.^1^ Moreover, studies have suggested that students make 47–77% of their career decisions by the end of pre-clerkship (years 1 and 2).^2^,^3^ To aid students in this regard, many Canadian medical schools, have focused on enhancing the pre-clerkship curriculum to include informative career direction experiences. This includes, but is not limited to lunchtime talks, mandatory observerships, and faculty career nights. Although such experiences may aid with career direction, they may not include all factors involved in making an informed career choice. Factors such as the types of interactions students seek between patients and healthcare professionals, and the desire for having continuity of care during future practice are not addressed.^4^–^7^ These elements may not be properly considered by medical students due to the lack of long-term patient-care and healthcare experiences available in the typical pre-clerkship curriculum.

To our knowledge, the current undergraduate curricula of Canadian medical schools offer few opportunities for students to engage in longitudinal patient care early on in their training. However, one program that exposes students to this form of care is the First Patient Program (FPP), a unique 18-month long program mandated within the undergraduate medical curriculum at Queen’s University. One of the main objectives of the program is to provide students with early clinical patient encounters during the pre-clerkship years with the goal of allowing students to experience the healthcare system from the patient’s perspective.^8^ In the FPP, first year medical students, at the beginning of the year, are randomly paired with chronically ill patients. The students then follow their patients on a journey through the healthcare system, attending at least three self-scheduled healthcare appointments with their patients over the 18-month period. During these appointments, which can include visits with non-MD healthcare professionals, students act as observers and reflect on their experiences through structured reports. A supervising physician, a family physician or other specialist, who is often the main healthcare provider for the patient, acts as a mentor for students, providing insight into patient care, and a glimpse into his/her specialty. The stated objective of the FPP is for students to gain an understanding of the impact of chronic diseases from a patient’s perspective, something that would otherwise not be possible for medical students given the lack of longitudinal experiences normally available in medical school.

Although career exploration was not an explicit objective of the FPP, we aimed to investigate whether the exposure of medical students to patients early in their training could provide guidance for career decisions as an unintended outcome of the program. We hypothesized that students with increased participation in such a program would derive more career guidance.

## Methods

### Study design

This was a cross-sectional study with the use of an online survey, which gathered qualitative and quantitative information. Each participant submitted and answered one survey containing four questions (Table 1). The Institutional Review Board at Queen’s University approved this study (#6013162).

### Participants

Queen’s University medical students from three consecutive cohorts, who had participated at least six-months in the FPP, were recruited to voluntarily participate in the study. An information sheet was provided prior to survey administration and consent was obtained as implied when the participant submitted the survey. Incomplete surveys were excluded from analysis.

### Thematic analysis

All responses to question four were analyzed using constant comparison methods, where the data were contrasted and compared for significant sentences and phrases. The open coding structure, computer aided thematic analysis software – nVIVO (v2.0, QSR International Pty. Ptd., Melbourne, Australia) was used for data organization and retrieval.

### Statistical analysis

Descriptive statistics were used to record the students’ year of study, the number of FPP appointments attended, and whether students believe the FPP had an impact on their career decision. Pearson’s Chi Square Test was used to compare the three cohorts’ responses to perceived impact of FPP on career decision. The Fischer’s Exact Test was applied to compare students who attended three or fewer FPP appointments to those with four or more in relation to career impact. All statistical analyses were performed with SPSS (v18.0, IBM Corporation, New York, United States). Statistical significance was set at an alpha level of 0.05.

## Results

Overall, 148 students completed the survey, with a response rate of 49%. Of the respondents, 130 attended three appointments or fewer at the time of the survey, while 18 attended four or more.

Twenty-eight (19%) students reported that the FPP impacted their personal choice regarding career directions, in contrast to 120 (81%) who indicated the FPP did not help with career guidance. Comparisons among the three cohorts did not reveal differences in response to whether FPP impacted career directions (p=0.685).

**Figure 1:**
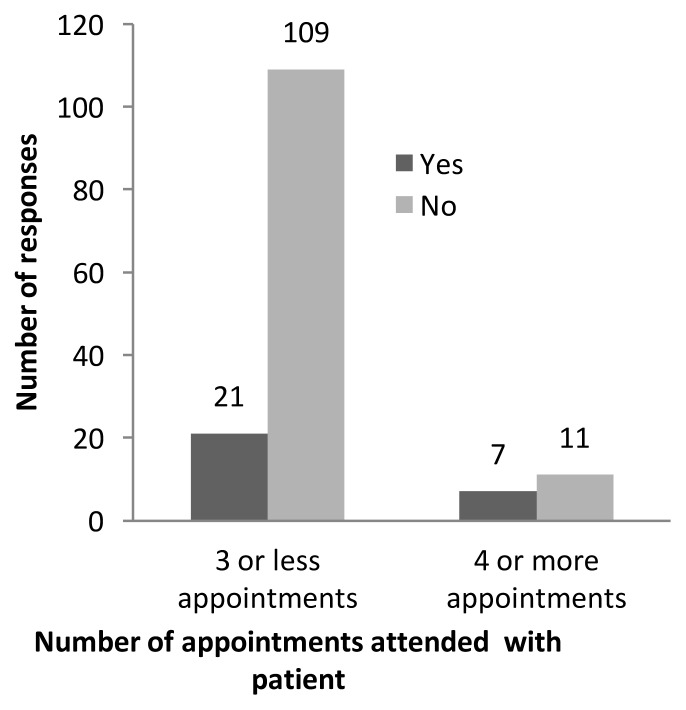
Number of appointments attended during the FPP and student reported FPP impact on career choices

Of the students who attended four or more appointments, 39% (7/18) responded “Yes” to having derived career guidance from the FPP, as compared to students who attended three or fewer appointments, of which 16% (21/130) answered “Yes” (p=0.021, Figure 1).

Thematic analysis of qualitative responses to the question “Was FPP as an influence on career direction?” revealed two common themes among students: 1) Primary focus of students was to learn more about a complex patient’s journey through the healthcare system; and 2) Although all experiences, including this program, help students guide their career choices, other experiences are more influential in this respect. Thematic breakdown and direct quotations are included in Table 2.

## Discussion

In the current Canadian medical curriculum, medical students have limited opportunity to follow patients over the long term. Recent reports revealed that long-term patient exposure in medical school could impart career guidance for students.^9^,^10^ In our study, we found that most students did not derive career direction from the FPP, which we attributed to several reasons highlighted in the thematic analysis results of the students’ responses. The first theme showed that students were overall well attuned to the FPP’s main objective – to gain an enhanced view of the healthcare system from the patient’s perspective. As a stated objective that was subsequently communicated through the duration of the program, students were cognizant of the program’s intended goals.

However, as career exploration was not explicitly stated as an intended objective, students may not have actively used the FPP as a source of career exploration. More explicit objectives and scaffolding within report templates centered on career exploration within the FPP may guide students’ experiences.

Moreover, the second theme revealed that a majority of students used alternative experiences such as observerships and clerkship rotations to guide their career choices. Self-scheduled observerships, within both medicine and surgery, have been shown to aid students in career decision making.^11^,^12^ Observerships are activities that have explicit goals of career exploration and are also self-scheduled by students to potentially align with their interests. The FPP was not designed to support career exploration: students were assigned to a patient and supervising physician regardless of specialty, and many healthcare visits were with non-MDs. For a longitudinal learning exercise to derive increased career direction experiences we believe asking for and accounting for students’ fields of interest when assigning patients and mentors may be helpful.

From this study we were able to take away several key points about the program’s impact and several ways the FPP can improve to potentially impart career direction experiences, an important objective of medical school. Some students could not attend enough FPP appointments due to time constraints stemming from other curricular obligations, while others were not paired with patients in the field that suited their interests. It may be helpful to ask students to consider career decision-making as part of their FPP experience, by determining the type of interaction they desire between patients and healthcare professionals, and whether continuity of care in their future practice is important.^4^–^7^ Additionally, mandating students to engage in more than three healthcare appointments may provide students with increased opportunity to observe their patients in their medical appointments, therefore providing more insight into specific medical disciplines. Improvements in all of these areas can help to further enhance the student experience within FPP.

The main limitation of our study is that we were not able to account for potential confounders like students’ prior experiences or pre-determined career interests. These factors may have altered how the program impacted our students, and hence the results of our study.

### Conclusion

Our study suggests that a program of longitudinal patient exposure that emphasizes the impact of chronic diseases from a patient’s perspective does not convey significant career direction to medical students, but further research is certainly recommended. The integration of explicit career learning objectives, requiring students to attend more appointments, and incorporating students’ personal interest when assigning patients and physicians may help future students derive career direction from such a program.

## Figures and Tables

**Table 1:** Survey Questions

Survey questions
Which year of medicine are you in? How many healthcare appointments were you able to attend with your First Patient through the duration of the First Patient Program? Has participating in the First Patient Program impacted your personal choices regarding career direction? (Yes/No) For question 3, if you answered “Yes”, please explain how. If you answered “No”, please elaborate on the response.

**Table 2:** Thematic analysis results

**Theme 1 - Primary focus of students was to learn more about a complex patient’s journey through the healthcare system**
Writing reflections recalled impact and motivating moments “The events especially sticks out because of all the writing and reflection that went with it, so I remember my thoughts well” Appreciation of the difficulties and frustrations of the medical system for patients “The focus of the program for me was the barriers that my patient faced in different components of the healthcare system” Reinforced importance of advocating for patients, and patient-centered care *“It reaffirmed my love of patient interaction and the importance of developing long-term, strong relationships with patients”*
**Theme 2 - Although all experiences, including this program, help students guide their career choices, other experiences are more influential in this respect**
Goal of the FPP was to assist in career selection not recognized by students “I don’t think that the FPP’s goal nor its outcome was to assist in career selection” Students had mixed reactions about the usefulness of FPP interactions in influencing their own future career direction “Attending such appointment are very educational, but do not generally give direction regarding the differences between different specialties” Experiences with clerkship and observership was more influential with career selection “I think my decision to pursue family medicine is more so influenced by my core rotation and speaking to other family physicians”
